# LncRNA H19-Derived miR-675-3p Promotes Epithelial-Mesenchymal Transition and Stemness in Human Pancreatic Cancer Cells by targeting the STAT3 Pathway

**DOI:** 10.7150/jca.44833

**Published:** 2020-06-06

**Authors:** Feng Wang, Long Rong, Zhengkui Zhang, Mingzhe Li, Ling Ma, Yongsu Ma, Xuehai Xie, Xiaodong Tian, Yinmo Yang

**Affiliations:** 1 Department of General Surgery, Peking University First Hospital, Beijing 100034, People's Republic of China.; 2 Department of Endoscopy Center, Peking University First Hospital, Beijing 100034, People's Republic of China.; 3 Department of Surgical Oncology, Peking University Ninth School of Clinical Medicine (Beijing Shijitan Hospital, Capital Medical University), Beijing 100038, People's Republic of China.

**Keywords:** pancreatic cancer, long noncoding RNA H19, miR-675-3p, STAT3 signaling pathway, EMT, stemness property

## Abstract

**Objective:** The functional role and mechanism of the long noncoding RNA (lncRNA) H19 in regulating human pancreatic cancer (PC) cell stemness and invasion have not been completely elucidated. This study aimed to evaluate the role of H19 in regulating the stemness, epithelial-mesenchymal transition (EMT), invasion and chemosensitivity of PC cells.

**Methods:** The sphere-forming ability was assessed using serum-free floating-culture systems. Chemosensitivity was evaluated via CCK-8 and flow cytometry assays *in vitro*. Migration and invasion were evaluated by transwell assays. The expression of stemness and EMT markers was detected by flow cytometry, qRT-PCR and western blot analyses. Xenograft initiation, growth and sensitivity were examined; Ki-67 nuclear staining intensity was evaluated by immunohistochemistry; and *in situ* apoptosis was evaluated by a TUNEL assay.

**Results:** H19 played an important role in maintaining PC cell stemness. Upregulated H19 expression in CAPAN-1 cells promoted tumor cell migration, invasion, EMT and chemoresistance. In contrast, downregulated H19 expression in PANC-1 cells yielded the opposite results. These effects were mediated by positively modulating the STAT3 pathway. Furthermore, SOCS5, an endogenous inhibitor of the STAT3 pathway, was a direct target of miR-675-3p, which was positively regulated by H19 in PC cells.

**Conclusions:** The H19/miR-675-3p signaling axis plays a critical role in maintaining the EMT process and stemness of PC cells by directly targeting SOCS5 to activate the STAT3 pathway. These data provide new insights into the oncogenic function of H19 in human PC and reveal potential targets for the development of optimal treatment approaches for this disease.

## Introduction

Pancreatic cancer (PC) is an aggressive malignancy with a poor prognosis and was reported to be the fourth leading cause of cancer-related death in the United States and the seventh leading cause of death worldwide in 2018 1. Despite recent and rapid improvements in surgical techniques and neoadjuvant and adjuvant chemotherapy, the overall 5-year survival rate of patients with this disease is less than 9%, and the survival rate has not improved significantly for several decades 2-4. Radical resection offers the greatest advantage for long-term survival. However, approximately 80-85% of patients are diagnosed with an advanced stage of the disease; consequently, radical resection is not feasible for most patients with PC 5,6. Therefore, a better understanding of the molecular events underlying PC development and progression is imperative for early diagnosis and the development of novel therapeutic strategies for this disease.

Long noncoding RNAs (lncRNAs) are a group of noncoding RNAs that contain mRNA-like transcripts that are longer than 200 ribonucleotides and lack open reading frames. Based on accumulating evidence, lncRNAs are involved in carcinogenesis, tumor growth and the development of distant metastases in various types of cancer. However, most lncRNAs are difficult to characterize because of their high turnover, poor conservation and differences in abundance, and only highly conserved, stable and abundant lncRNAs are candidates to serve as potential biomarkers and therapeutic targets in cancers 7,8. H19 is an imprinted lncRNA that is exclusively transcribed from the paternal allele of the H19 gene located on human chromosome 11p15.5. Abnormal H19 expression has been observed in many cancers. In most malignancies, H19 plays a role as an oncogene involved in cell proliferation, cell cycle regulation, apoptosis, migration, invasion, and stemness, as well as the promotion of tumorigenesis in different types of cancers 9-12. As shown in our previous study, H19 is expressed at high levels in human PC tissues and is related to tumor differentiation; it functions as an oncogene in PC cells by promoting cancer cell proliferation by upregulating E2F-1 7. However, the functional role and mechanism of H19 in regulating the stemness and invasion of PC cells have not been completely elucidated.

The mechanisms by which lncRNAs regulate their target genes include modulation of chromatin remodeling, degradation of mRNAs and expression of microRNAs (miRNAs). The cancer-promoting effects of H19 are largely mediated by microRNA-675 (miR-675), a short noncoding RNA transcribed from the first exon of H19 13-15. In the process of the transcriptional expression of H19, a primary miRNA precursor of miR-675 is simultaneously encoded. However, the relationship between H19 and miR-675 remains controversial. A positive correlation between these two noncoding RNAs was reported in gastric and colorectal cancer tissues 14,15, but in our previous study, a negative correlation was found between H19 and miR-675-5p expression 16. Notably, miR-675-5p executes the functions of H19 by targeting the transcription factor E2F-1 and regulating the proliferation of PC cells 16. However, the correlation between H19 and miR-675-3p expression in human PC remains unclear.

Based on our previous results showing that H19 correlates with the tumor differentiation of human PC, we speculated that H19 may play essential roles in regulating the stemness, epithelial-mesenchymal transition (EMT), invasion and chemosensitivity of PC cells. In this study, we demonstrated that H19 played an important role in maintaining the stemness of PC cell lines, and the elevated H19 expression observed in PC cells was associated with the EMT process, increased migration and invasion, and chemoresistance. These effects were mediated by activation of the STAT3 pathway. Furthermore, SOCS5, an endogenous inhibitor of the STAT3 pathway, was a direct target of miR-675-3p, and the expression of miR-675-3p was positively regulated by H19 in PC cells. miR-675-3p was required for the changes in both SOCS5 expression and STAT3 activity mediated by H19 modulation. Based on these data, the H19/miR-675-3p signaling axis plays a critical role in maintaining the stemness and EMT process of PC cells by directly targeting SOCS5 to activate the STAT3 pathway.

## Materials and methods

### Reagents

Fetal bovine serum (FBS), RPMI-1640 and Dulbecco's modified Eagle's medium (DMEM) were purchased from Gibco (Carlsbad, CA, USA). Gemcitabine was obtained from Eli Lilly (Indianapolis, IN, USA). Antibodies against STAT3, phospho-STAT3, E-cadherin and Snail were purchased from Cell Signaling Technology (Beverly, MA, USA). Antibodies against ALDH1A1, Ki67, and N-cadherin were purchased from Abcam (Cambridge, MA, USA). The antibody against SOCS5 was purchased from OriGene Technologies (Rockville, MD, USA). The anti-β-actin antibody used in this study was purchased from Medical & Biological Laboratories Co., Ltd. (Nagoya, Japan). Recombinant human IL-6 was obtained from PeproTech (Rocky Hill, NJ, USA), and AG490 was purchased from Selleckchem (Houston, TX, USA).

### Cell culture and transfection

The PC cell lines CAPAN-1 and PANC-1 and the retroviral packaging cell line 293T were purchased from the American Type Culture Collection (ATCC, Manassas, VA, USA). The cell lines were cultured in RPMI-1640 or DMEM supplemented with 10% FBS, penicillin (100 U/ml) and streptomycin (100 µg/ml). All cell lines were maintained at 37°C in a humidified, 5% CO_2_ atmosphere.

shRNAs targeting H19 (shH19-1, CCCACAACATGAAAGAAAT and shH19-2, GACGTGACAAGCAGGACAT) and a negative control with a scrambled sequence were designed and constructed into GV248 lentiviral vectors by GeneChem (Shanghai, China). The full-length H19 sequence was subcloned into the lentiviral vector Lenti-GFP (GeneChem). The lentiviruses were produced by transfecting 293T cells with plasmids using a packaging mix (GenePharma, Shanghai, China). Cells were transfected and then selected with puromycin according to the manufacturer's instructions. miRNA mimics and inhibitors were purchased from RiboBio (Guangdong, China), and the transfections were performed with Lipofectamine 2000 (Invitrogen, Carlsbad, CA, USA) according to the manufacturer's instructions.

### Quantitative reverse transcription-polymerase chain reaction (qRT-PCR)

Total RNA was extracted from the indicated cell lines using TRIzol (Invitrogen) and reverse transcribed using the ReverTra Ace® qPCR RT Kit (Toyobo, Osaka, Japan). qRT-PCR was conducted in an ABI 7500 system (Applied Biosystems, Foster City, CA, USA) using cDNAs, SYBR Green PCR Master Mix (Toyobo) and specific primer pairs according to the manufacturer's protocol. The ALDH1A1 primer was designed and constructed by QIAGEN (Hilden, Germany). The H19 primer, the U6 primer and the miR-675-3p primer were designed and constructed by RiboBio (Guangdong, China). GAPDH and U6 were used as endogenous controls to normalize the mRNA and miRNA levels, respectively. The relative gene expression levels were quantified in triplicate, and the cycle threshold (CT) value for each gene was normalized to the CT value for GAPDH or U6. The fold changes in expression relative to a reference sample were calculated using the 2^-△△^Ct method with ABI 7500 software v2.0.6 (Applied Biosystems). The primer sequences are as follows: GAPDH, 5'-ACGGATTTGGTCGTATTGGG-3' and 5'-TGATTTTGGAGGGATCTCGC-3'; Snail, 5'-TGCCCTCAAGATGCACATCCGA-3' and 5'-GGGACAGGAGAAGGGCTTCTC-3'; E-cadherin, 5'-GCCTCCTGAAAAGAGAGTGGAAG-3' and 5'-TGGCAGTGTCTCTCCAAATCCG-3'; N-cadherin, 5'-TTTGATGGAGGTCTCCTAACACC-3' and 5'-ACGTTTAACACGTTGGAAATGTG-3'; Oct-4, 5'-CTTGAATCCCGAATGGAAAGGG-3' and 5'-GTGTATATCCCAGGGTGATCCTC-3'; and SOCS5, 5'-GTGCCACAGAAATCCCTCAAA-3' and 5'-TCTCTTCGTGCAAGTCTTGTTC-3'.

### Western blot analysis

Cells were lysed in cold RIPA buffer that had been freshly supplemented with 1 mmol/l phenylmethylsulfonyl fluoride (PMSF), a phosphatase inhibitor cocktail (KeyGEN Biotech, Nanjing, China) and a protease inhibitor cocktail (Amresco, Houston, TX, USA). Total protein concentrations were measured with the BCA Protein Assay Kit (Thermo Scientific, Rockford, IL, USA). Then, the proteins were separated on 10% SDS-PAGE gels and transferred to nitrocellulose (NC) membranes. Membranes were blocked with 5% nonfat dry milk in Tris-buffered saline containing 0.1% Tween 20, incubated with the appropriate primary antibodies, and then incubated with the appropriate HRP-conjugated secondary antibodies. Peroxidase activity was detected with Immobilon Western Chemiluminescence HRP Substrate (Millipore, Billerica, MA, USA). β-actin served as a loading control.

### Transwell assay

We seeded cells (3.0 × 10^4^ cells/well) suspended in DMEM or RPMI-1640 into the upper chamber of 24-well Corning® FluoroBlok^TM^ Cell Culture Inserts (NY, USA) to detect cell migration. The lower chamber was filled with DMEM or RPMI-1640 supplemented with 10% FBS, which served as a chemoattractant. The cells were cultured for 48 h and were then counted under an inverted fluorescence microscope. We seeded cells (5.0 × 10^4^ cells/well) suspended in DMEM or RPMI-1640 into the upper chamber of 24-well Corning® FluoroBlok^TM^ Cell Culture Inserts that had been precoated with a 1:7 dilution of Matrigel Matrix (Corning) according to the manufacturer's instructions to detect cell invasion. The lower chamber was filled with DMEM or RPMI-1640 supplemented with 10% FBS, which served as a chemoattractant. The cells were cultured for 48 h before being counted under an inverted fluorescence microscope.

### CCK-8 assay

Cell viability was detected using a Cell Counting Kit-8 (CCK-8, Dojindo Laboratories, Kumamoto, Japan). Cells were plated in 96-well plates at a density of 3 × 10^3^ cells/well. The experiment was performed in triplicate. After the cells were exposed to gemcitabine for 72 h, 100 μl of serum-free cell culture medium containing 10 μl of WST-8 reagent was added to each well, and the plates were incubated under standard conditions for 1 h. The optical absorbance of each well was measured at 450 nm and 630 nm in a microplate reader (Bio-Rad Laboratories, Hercules, CA, USA).

### CD24+CD44+ESA+ cell subfractionation assay

We used the following antibodies to detect the expression of cancer stem cell (CSC) markers: anti-CD44-PE, anti-CD24-APC, anti-epithelial-specific antigen (ESA)-PE-Vio770, IgG1-PE, IgG1-APC, and IgG1-PE-Vio770 (Miltenyi Biotec, Bergisch Gladbach, Germany). Cells were harvested by digestion with trypsin without EDTA to produce a single-cell suspension. The cells were pelleted by centrifugation, washed twice with phosphate-buffered saline (PBS) and then incubated with the appropriate antibody in the dark for 10 min at 4°C. The appropriate isotype control antibodies were used at the same concentrations according to the manufacturer's instructions. After two washes with PBS, the samples were resuspended in 500 μl of PBS and analyzed with a BD FACSCalibur flow cytometer (BD Biosciences, San Jose, CA, USA) within 1 h.

### Apoptosis assay

We harvested cells in the exponential growth phase by incubating them with trypsin without EDTA and seeded them into 6-well plates to detect apoptosis. Once the cells adhered to the plate, the experimental group was treated with gemcitabine (0.01 μg/ml for CAPAN-1 cells and 50 μg/ml for PANC-1 cells). The cells were then cultured for 72 h, and an Annexin V-PE/7-AAD Apoptosis Assay Kit (KeyGEN BioTECH) was subsequently used to measure the percentage of apoptotic cells. Apoptosis was analyzed using a BD FACSCalibur flow cytometer within 1 h.

### Dual-luciferase assay

Using bioinformatics analyses, we identified a fragment of SOCS5 as a miR-675-3p target. The 3'-UTR of the human SOCS5 mRNA was constructed using synthetic oligonucleotides and a sequence containing the wild-type or a mutant SOCS5-binding site was cloned into the psi/check2 vector (Syngentech Co., LTD., Beijing, China). PANC-1 cells were seeded into 96-well plates (10,000 cells per well), and the psi/check2-SOCS5-3'-UTR vectors containing wild-type or mutated miR-675-3p-binding sites were cotransfected with a miR-675-3p mimic into PANC-1 cell lines. Then, 48 h after transfection, the firefly and Renilla luciferase activities were examined using the Dual-Luciferase Assay System (Promega, Madison, WI, USA). The relative expression of firefly luciferase activity was normalized to that of Renilla luciferase activity.

### Mouse xenograft model

Equal numbers of H19-overexpressing cells (2 × 10^6^ cells) and negative control cells were resuspended in 100 μl of PBS and subcutaneously injected into the bilateral axillary fossae of six-week-old nude mice (BALB/c-nu). The tumor volume was calculated using the formula V = 0.5ab^2^ (a, the longest tumor axis; b, the shortest tumor axis). After 15 days, the mice received intraperitoneal injections of gemcitabine (50 mg/kg) or an equal volume of vehicle (PBS) every three days. At the end of the study, all mice were sacrificed, and the tumors were excised. The animal studies were reviewed and approved by the Ethics Committee for Animal Use and Care at Peking University First Hospital (Approval Number: J201920A).

### Immunohistochemical staining

Xenograft tissues collected from the nude mice were fixed with 4% paraformaldehyde, embedded in paraffin, and then deparaffinized and rehydrated. After antigen retrieval, the sections were immersed in 0.3% hydrogen peroxide for 15 min and then incubated with an anti-Ki-67 antibody overnight at 4°C. Next, the sections were incubated with an HRP-conjugated secondary antibody and observed under a microscope. Five fields were randomly selected to calculate the percentage of Ki-67-positive staining.

### TUNEL assay

Apoptosis in the xenograft tissue was detected using a TUNEL *in situ* apoptosis detection kit (KeyGEN BioTECH) according to the instructions provided in the package insert. After each tissue sample was observed under a microscope, five fields were randomly selected to calculate the percentage of TUNEL-positive cells.

### Statistical analysis

All data are presented as the means and standard deviation (SD) unless stated otherwise, and all data were analyzed with SPSS version 13.0 software (SPSS, Chicago, IL, USA) and graphed by GraphPad Prism 5 (La Jolla, CA, USA). Comparisons between two groups were performed with a paired or an unpaired two-tailed Student's t-test. Statistical significance was defined as P < 0.05 (* P < 0.05, ** P < 0.01, *** P < 0.001, and **** P < 0.0001). The results are presented as the means ± SD from at least three separate experiments.

## Results

### A substantial increase in H19 expression was detected in the PC stem cell spheroids, and H19 regulated the migration, invasion and EMT of PC cells

**Figure 1:**
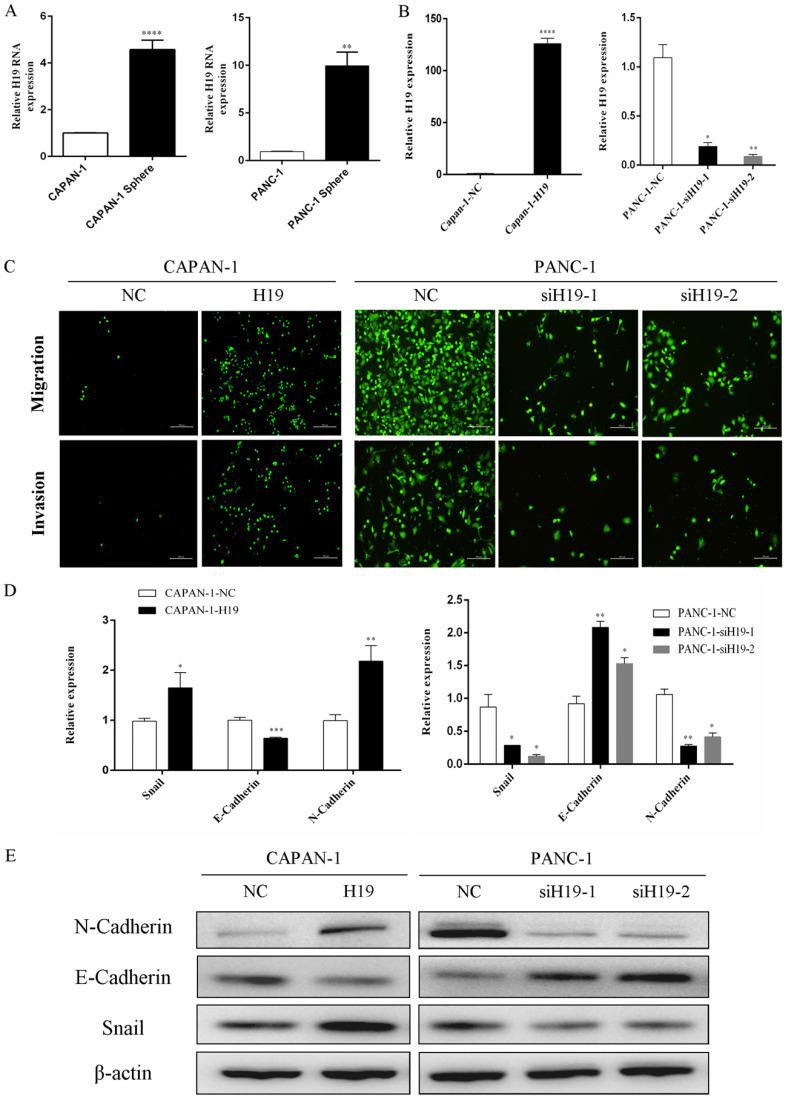
** H19 expression was increased in tumorspheres, and H19 promoted EMT of PC cells. (A)** qRT-PCR revealed a substantial increase in H19 expression in tumorspheres compared with that in the respective parental CAPAN-1 and PANC-1 cells. **(B)** H19 expression was detected in CAPAN-1 cells overexpressing H19 and in PANC-1 cells expressing H19-shRNAs using qRT-PCR. **(C)** Cell migration and invasion were assessed using transwell assays. **(D)** The mRNA expression of EMT markers was detected using qRT-PCR. **(E)** The protein levels of EMT markers were detected using western blotting. The qRT-PCR data are presented as the means ± SD. * P < 0.05, ** P < 0.01, *** P < 0.001, and ****P < 0.0001.

As shown in our previous study, compared with that in normal human pancreatic ductal cells, H19 expression is remarkably upregulated in PC cell lines, especially in BxPC-3, T3M4 and PANC-1 cells 7. PANC-1 cells (with high H19 levels) and CAPAN-1 cells (with relatively low H19 levels) were used to examine the functions of H19 in the current study. First, we isolated tumorspheres from both CAPAN-1 and PANC-1 cells using a serum-free floating-culture system, which has been shown to contain a significantly high percentage of the CD44+CD24+ESA+ population and can differentiate stem-like properties 17. The expression of H19 was detected and compared between the tumorspheres and their respective paternal cell lines. Both CAPAN-1 and PANC-1 tumorspheres displayed substantially increased H19 expression (Figure 1A). Next, we investigated the functions of H19 in PC cells, focusing on migration, invasion and EMT. Lentiviruses containing the full-length H19 sequence were used to overexpress H19 in CAPAN-1 cells (CAPAN-1-H19), and H19-silenced cells were established using PANC-1 cells (PANC-1-siH19-1 and PANC-1-siH19-2), as shown in Figure 1B. The Boyden chamber technique and Matrigel assays were used to determine the percentages of migrating and invading cells. Upregulation of H19 significantly increased the migration and invasion of CAPAN-1 cells, while downregulation of H19 significantly inhibited the migration and invasion of PANC-1 cells (Figure 1C). Then, EMT markers were detected using qRT-PCR and western blotting, and the results indicated that upregulation of H19 was associated with increased levels of the mesenchymal phenotypic marker N-cadherin and the transcription factor Snail but was associated with downregulated E-cadherin expression in CAPAN-1 cells. Downregulation of H19 in PANC-1 cells produced the opposite results (Figure 1D and E). Based on these data, H19 is involved in CSC spheroid formation and may regulate EMT, migration and invasion of PC cells.

### H19 is involved in maintaining stemness and regulating the chemosensitivity of PC cells *in vitro* and *in vivo*

**Figure 2:**
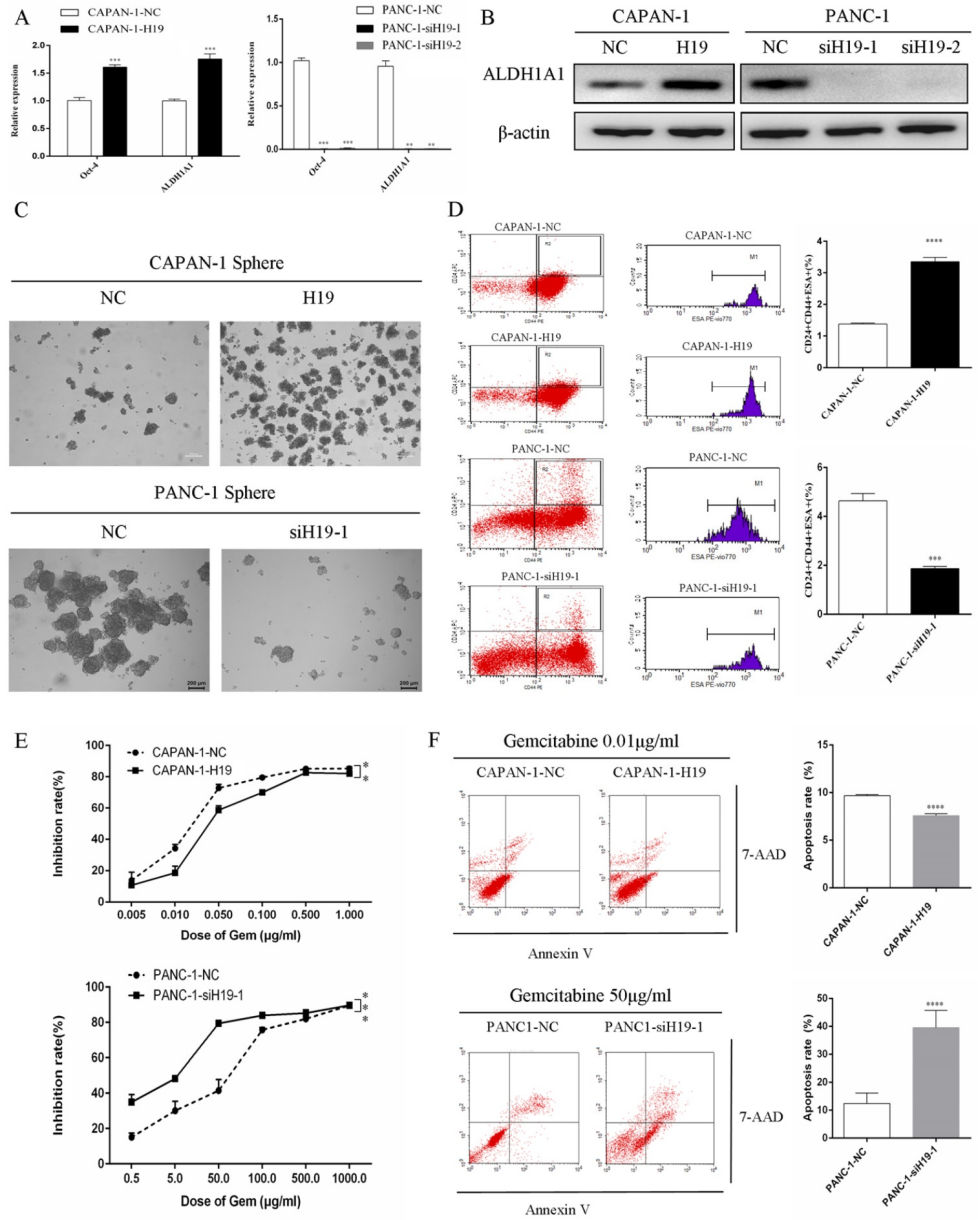
** Effects of H19 on the maintenance of stemness and chemosensitivity of PC cells. (A)** The mRNA expression of CSC markers was detected using qRT-PCR. **(B)** The levels of the ALDH1A1 protein were determined using western blotting. **(C)** The tumorsphere-forming assay revealed positive correlations of both the numbers and diameters of tumorspheres with H19 expression. **(D)** Flow cytometry analysis showed the percentages of the CD24+CD44+ESA+ cell subfraction. **(E)** The CCK-8 assay revealed the chemosensitivity of each cell clone to gemcitabine. **(F)** Flow cytometry analysis showed the apoptosis rate of each cell clone after treatment with gemcitabine for 72 h. The data are presented as the means ± SD. ** P < 0.01, *** P < 0.001, and **** P < 0.0001.

**Figure 3:**
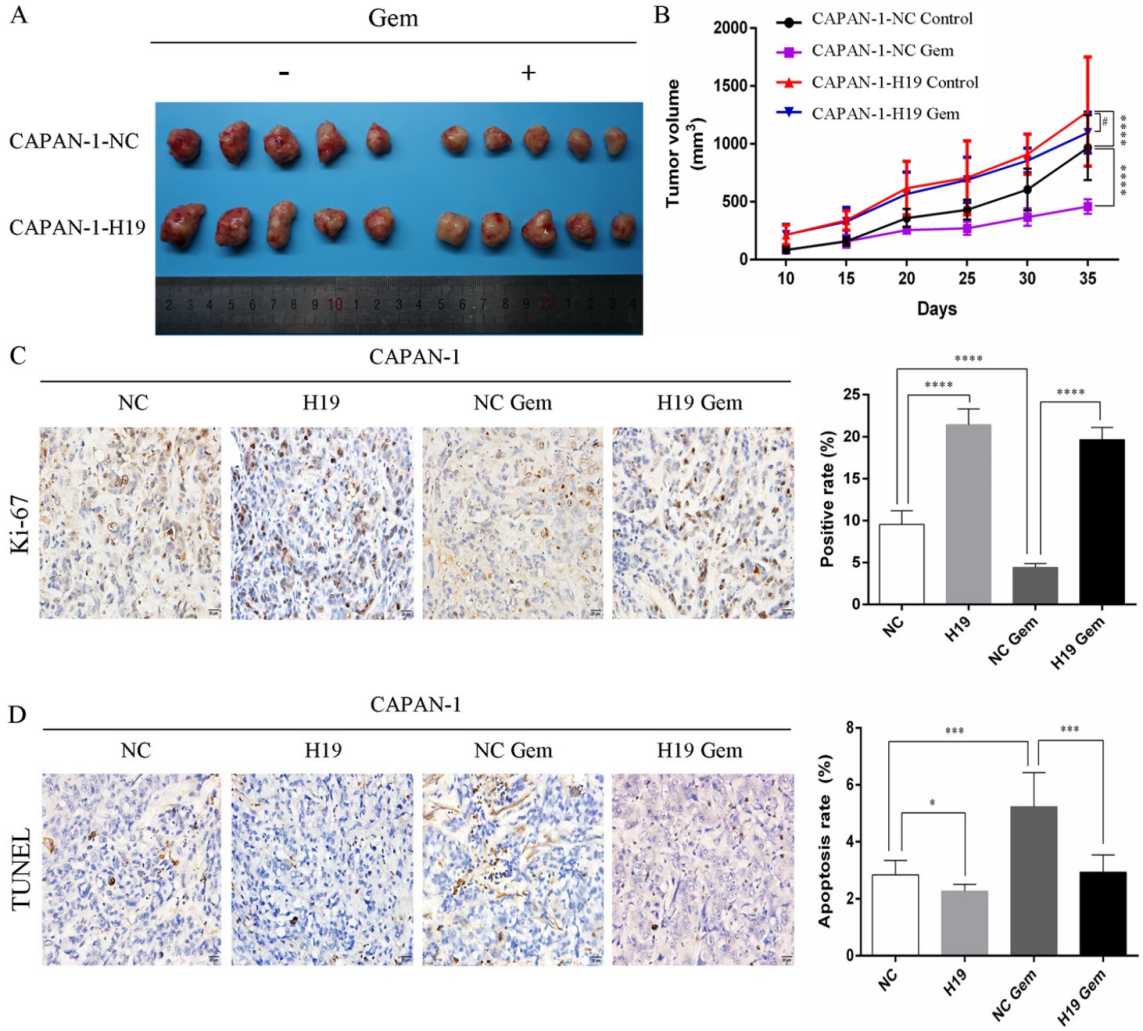
** Effects of H19 on tumor growth and chemosensitivity in nude mouse xenograft models. (A)** Xenograft tumors were removed from mice implanted with different cells treated with or without gemcitabine. **(B)** *In vivo* tumor growth curves for each group. **(C)** The Ki-67 nuclear staining intensity for each group was evaluated using immunohistochemistry. **(D)** The apoptosis rate in tumors was evaluated using a TUNEL assay. The data are presented as the means ± SD. # P > 0.05, * P < 0.05, *** P < 0.001, and **** P < 0.0001.

The expression of ALDH1 and Oct-4, the tumorsphere-forming ability, and the proportion of CD24+CD44+ESA+ cells were detected to assess the ability of H19 to maintain the stemness of PC cells. H19 overexpression was associated with significantly increased levels of ALDH1A1 and Oct-4 in CAPAN-1 cells, while downregulation of H19 significantly decreased ALDH1A1 and Oct-4 expression in PANC-1 cells (Figure 2A and B). Inverted fluorescence microscopy revealed significant increases in both the numbers and diameters of tumorspheres after upregulation of H19 expression in CAPAN-1 cells, while fewer and smaller tumorspheres were observed in PANC-1 cells after downregulation of H19 (Figure 2C). The percentages of the CD24+CD44+ESA+ cell subfraction were measured using flow cytometry, and upregulation of H19 significantly increased the percentage of the CD24+CD44+ESA+ cell subfraction in CAPAN-1 cells, while downregulation of H19 significantly decreased the percentage in PANC-1 cells (Figure 2D). Consistent with these findings, upregulation of H19 in CAPAN-1 cells was associated with decreased chemosensitivity to gemcitabine and a lower apoptosis rate induced by the same concentration of gemcitabine. However, the chemosensitivity to gemcitabine and the apoptosis rate were significantly increased after inhibition of H19 in PANC-1 cells (Figure 2E and F). CAPAN-1 cells with or without H19 overexpression were used to establish xenograft models in nude mice to verify the oncogenic function of H19 in PC cells *in vivo*. CAPAN-1-H19 cells grew faster and were less sensitive to gemcitabine therapy than control CAPAN-1 cells (Figure 3A and B). Immunohistochemical staining for Ki-67 and the TUNEL assay produced consistent results (Figure 3C and D). Thus, H19 is involved in maintaining the population of CSCs and regulates the sensitivity of PC cells to gemcitabine chemotherapy *in vitro* and *in vivo*.

### H19 regulates SOCS5/STAT3 signaling to promote the malignant behavior of PC cells

**Figure 4:**
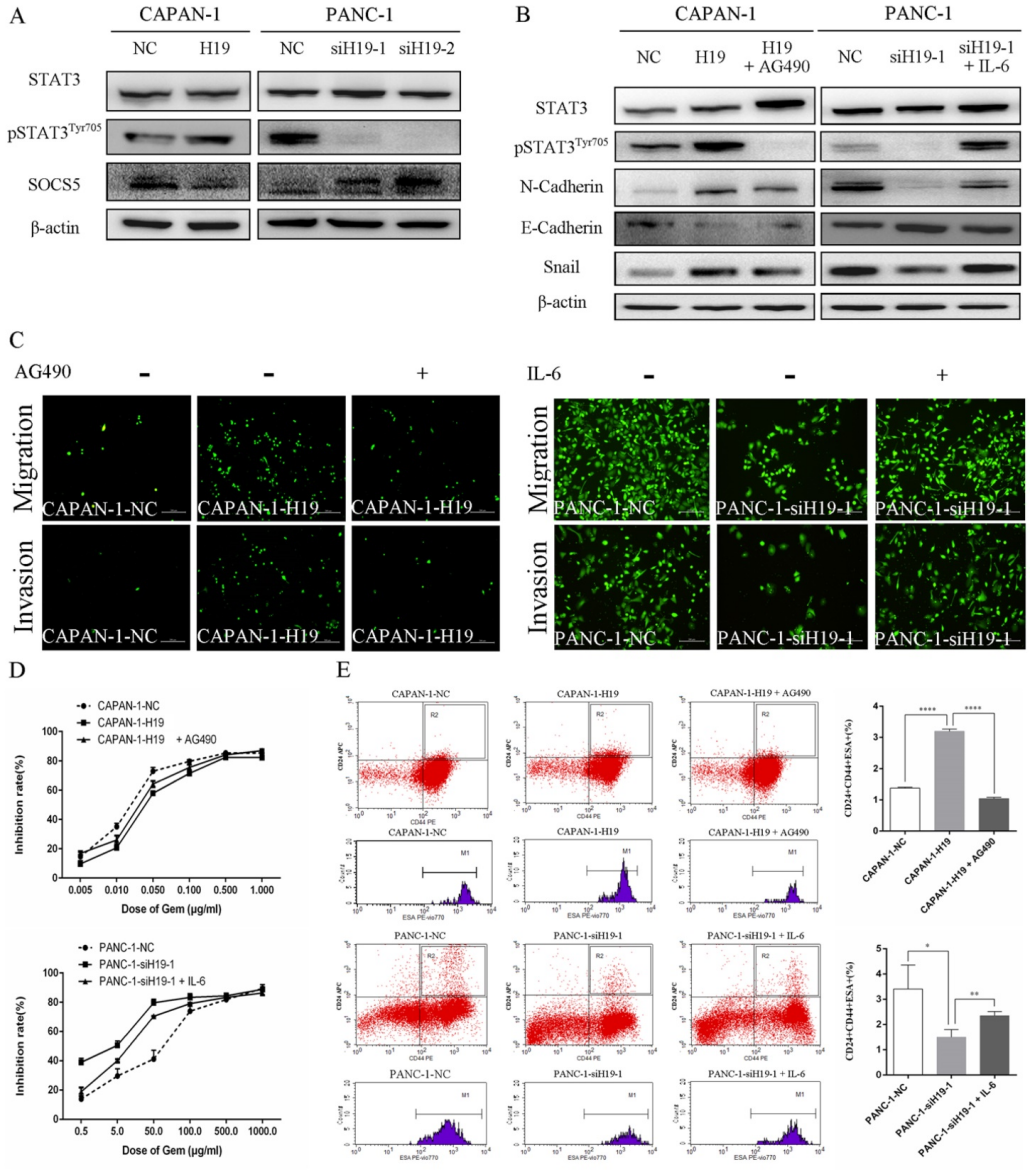
** H19 enhanced the malignant behaviors of PC cells by regulating SOCS5/STAT3 signaling. (A)** The levels of pSTAT3^Tyr705^ and SOCS5 in each cell clone were detected using western blotting. **(B)** The levels of pSTAT3^Tyr705^ and EMT markers in each cell clone were detected using western blotting. AG490 and IL-6 were used to modulate STAT3 activity. **(C)** Cell migration and invasion were examined using transwell assays. AG490 and IL-6 were used to modulate STAT3 activity. **(D)** The CCK-8 assay revealed the effect of AG490- or IL-6-induced changes in STAT3 activity on the chemosensitivity of each cell clone to gemcitabine. **(E)** Flow cytometry analysis showed the effects of AG490- or IL-6-induced changes in STAT3 activity on the CD24+CD44+ESA+ stem cell subfraction in each cell clone. The data are presented as the means ± SD. * P < 0.05, ** P < 0.01, and **** P < 0.0001.

The activity of STAT3 signaling and the expression of SOCS5, a member of the SOCS protein family that negatively regulates JAK2/STAT3 signaling, were detected using western blotting to identify the underlying oncogenic mechanisms of H19 in PC cells. H19 overexpression was associated with increased levels of pSTAT3^Tyr705^ and decreased levels of SOCS5, while downregulation of H19 exerted the opposite effects (Figure 4A). Moreover, the STAT3 signaling inhibitor AG490 and activator IL-6 were used to regulate the activity of the STAT3 signaling pathway. As shown in Figure 4B, AG490 reversed the effects of H19 overexpression on inducing STAT3 phosphorylation and the dysregulation of N-cadherin, E-cadherin, and Snail expression in CAPAN-1 cells. Conversely, IL-6 exerted the opposite effects on PANC-1 cells. Furthermore, modulation of STAT3 activity by AG490 and IL-6 restored the H19 dysregulation-induced changes in cell migration and invasion (Figure 4C), the chemosensitivity to gemcitabine (Figure 4D), and the percentage of the CD24+CD44+ESA+ cell subfraction (Figure 4E) in PC cell lines. In summary, H19 functions as an oncogene in PC cells by regulating SOCS5/STAT3 signaling.

### H19 regulates SOCS5/STAT3 signaling through cotranscription with miR-675-3p, and SOCS5 is a direct target of miR-675-3p

**Figure 5:**
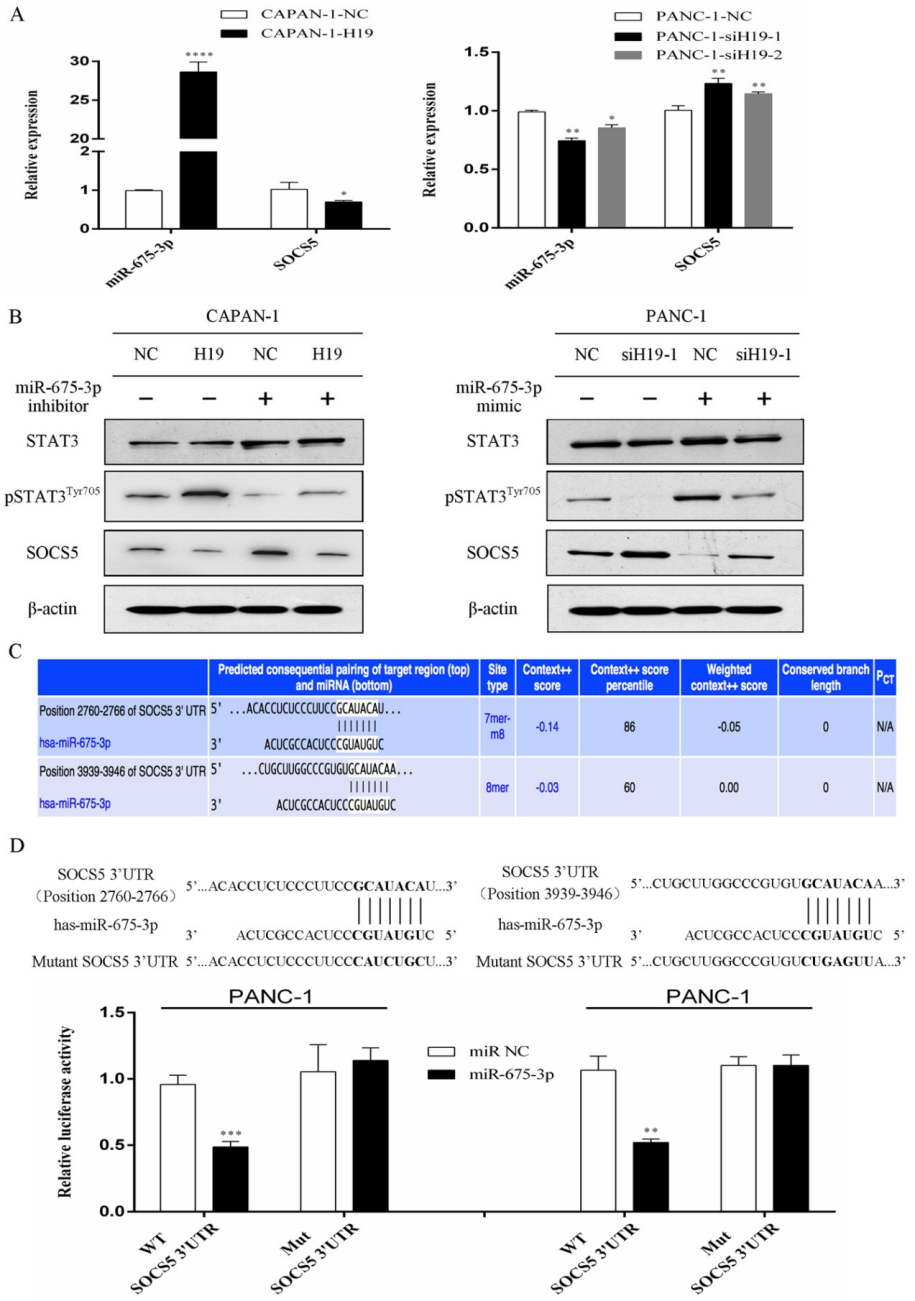
** H19 regulates SOCS5/STAT3 signaling by directly targeting SOCS5 via H19-derived miR-675-3p. (A)** The expression of miR-675-3p was positively correlated with H19 expression, while SOCS5 expression was negatively correlated with H19 expression in PC cell lines. **(B)** The effect of miR-675-3p on the modulation of pSTAT3^Tyr705^ and SOCS5 levels by H19. **(C)** SOCS5 was identified as a potential target gene of miR-675-3p using computer-aided miRNA target prediction programs. The nucleotide sequences of the target miRNA-binding sites in the SOCS5 3'-UTR are shown. **(D)** Luciferase assays confirmed that miR-675-3p binds to the wild-type 3'-UTR of SOCS5. The data are presented as the means ± SD. * P < 0.05, ** P < 0.01, *** P < 0.001, and **** P < 0.0001.

The expression levels of H19 were positively correlated with miR-675-3p expression in PC cells (Figure 5A). Furthermore, modulation of miR-675-3p expression by transfecting cells with a miR-675-3p inhibitor or miR-675-3p mimic rescued the changes in STAT3 phosphorylation and SOCS5 expression caused by H19 up/downregulation (Figure 5B), and miR-675-3p expression was negatively correlated with SOCS5 expression (Figure 5A and B). Potential target genes of miR-675-3p were searched using computer-aided miRNA target prediction programs, and SOCS5 was a potential candidate target gene of miR-675-3p because a binding site for this miRNA was found to be located within the 3'-UTR of SOCS5 (Figure 5C). We subcloned 3'-UTR SOCS5 fragments containing wild-type (SOCS5-WT) and mutant (SOCS5-MUT) miR-675-3p-binding sites into a psi/check2 vector to confirm whether miR-675-3p directly targeted SOCS5. SOCS5-WT or MUT-3'-UTR vectors were cotransfected into PANC-1 cells with the miR-675-3p mimic. As expected, the relative luciferase activity of the SOCS5-WT vector, but not the SOCS5-MUT vector, was significantly reduced in miR-675-3p-overexpressing PANC-1 cells (Figure 5D). Based on these data, H19/miR-675-3p regulates SOCS5/STAT3 signaling through the direct targeting of SOCS5 by miR-675-3p.

## Discussion

In our previous studies, H19 was overexpressed in both PC tissues and cell lines. H19 closely correlates with the degree of tumor differentiation and promotes PC cell proliferation by modulating the expression of E2F-1, a direct target of H19-derived miR-675-5p 7,16. In the current study, we mainly focused on the possible roles of H19 in regulating the stemness, EMT, invasion and chemosensitivity of PC cells. H19 was involved in maintaining the stemness of PC cells, and H19 overexpression in PC cells was associated with the EMT process and increased migration, invasion, and chemoresistance. These effects were at least partially mediated by STAT3 phosphorylation, which was induced by a decrease in the expression of SOCS5, a direct target of H19-derived miR-675-3p.

As shown in our previous study, H19 expression correlates with tumor differentiation in human PC, and H19 is expressed at substantially higher levels in poorly differentiated tumors than in well-differentiated tumors; in addition, poorly differentiated tumors exhibit more severe malignant phenotypes 7. Consistent with these findings, Yoshimura et al. 18 recently reported that H19 overexpression correlates with poorly differentiated cell types and has important roles in PC metastasis. Moreover, Sasaki et al. 19 reported that H19 played critical roles in the CSC self-renewal and cell adhesion of PC cells that led to invasion and metastasis, which is partially confirmed by our current research. However, in their study Sasaki et al. 19 also showed that H19 did not contribute to the expression of stemness-markers (including Oct-4), and H19 was negatively correlated to CD24, which appeared paradoxical to the observation that H19 was involved in sphere formation and invasion of PC cells. In the present study, we confirmed that high H19 expression was involved in maintaining the stemness of PC cells. Meanwhile, the expression of the stemness markers ALDH1A1 and Oct-4, as well as the proportion of CD24+CD44+ESA+ cells, were positively correlated with H19 expression in both PANC-1 and CAPAN-1 cell lines. Based on our findings, H19 overexpression in PC cells increased spheroid formation, EMT, migration and invasion, and H19 expression was correlated with the sensitivity of PC cells to gemcitabine chemotherapy *in vitro* and *in vivo*. Thus, H19 represents an essential oncogene contributing to the EMT process and maintaining the stemness of human PC cells.

The molecular mechanisms by which H19 regulates the EMT process and the maintenance of stemness remain unclear. Ma et al. 20 reported that H19 promotes PC metastasis by attenuating the suppressive effects of let-7 on its target, HMGA2, to mediate EMT. As shown in a recent study by Shima et al. 21, high H19 expression is associated with the stem cell phenotype in ALDH1-positive breast cancer, and H19 is associated with several genes such as miR-103, miR-107, let-7, miR-29b-1, and Trx, which are reported to regulate CSCs. In the current study, H19 expression was positively correlated with activation of STAT3 signaling in PC cells, which has been reported to be involved in tumor initiation, stemness maintenance and chemotherapy resistance 22-24. Approaches targeting STAT3 and its upstream regulators represent an emerging strategy for the treatment of human PC 25. SOCS5 is a member of the SOCS family that plays a vital role in suppressing JAK2/STAT3 signaling pathway activation 26. In this study, H19 expression was negatively correlated with SOCS5 expression but positively correlated with STAT3 activation. Moreover, modulation of STAT3 activity by either AG490 or IL-6 rescued the effects of H19 on EMT, migration, invasion, and chemosensitivity, as well as the CSC proportion, of PC cells. Collectively, it is possible that the effects of H19 on PC EMT and stemness are mediated by the regulation of the SOCS5/STAT3 signaling axis.

Importantly, miR-675 is a noncoding RNA transcribed from the first exon of H19 and performs many of the important biological functions of H19 13-15. In the present study, we confirmed the positive correlation between H19 and miR-675-3p expression in PC cell lines. Additionally, miR-675-3p was required for the changes in both SOCS5 expression and STAT3 activity mediated by H19 modulation. Furthermore, we are the first group to show that SOCS5 is a direct target of miR-675-3p in PC cells. Based on these data, we concluded that H19 represents an essential oncogene contributing to the EMT process and stemness maintenance in human PC cells by regulating STAT3 phosphorylation via the H19-derived miR-675-3p-SOCS5 axis.

In conclusion, we discovered a novel mechanism by which H19 activates the STAT3 pathway to promote EMT, chemoresistance and stemness maintenance in human PC cells. To the best of our knowledge, this report is the first to describe the role of the H19-derived miR-675-3p-SOCS5-pSTAT3^Tyr705^ axis in the modulation of STAT3 pathway activation in human PC cells. These data provide new insights into the oncogenic functions of H19 in human PC and reveal potential targets for the development of optimal treatment approaches for this disease.

## Abbreviations

lncRNA: long noncoding RNA
miRNAs: microRNAs
PC: pancreatic cancer
EMT: epithelial-mesenchymal transition
CSC: cancer stem cell
STAT3: signal transducers and activators of transcription 3
SOCS5: suppressor of cytokine signaling 5
qRT-PCR: quantitative reverse transcription-polymerase chain reaction
CCK-8: Cell Counting Kit-8
